# “You Could Tell I Said the Wrong Things”: Constructions of Sexual Identity Among Older Gay Men in Healthcare Settings

**DOI:** 10.1177/10497323211050373

**Published:** 2021-12-07

**Authors:** Hannah Kia, Travis Salway, Ashley Lacombe-Duncan, Olivier Ferlatte, Lori E. Ross

**Affiliations:** 1 School of Social Work, 8166 The University of British Columbia, Vancouver, BC, Canada; 2 Faculty of Health Sciences, 1763 Simon Fraser University, Burnaby, BC, Canada; 3 School of Social Work, 1259 University of Michigan, Ann Arbor, MI, USA; 4 École de Santé Publique, 5622 Université de Montréal, Montreal, QC, Canada; 5 Centre de Recherche en Santé Publique, Université de Montréal et CIUSSS Du Centre-Sud-de-l’Île-de-Montréal, Montreal, QC, Canada; 6 Dalla Lana School of Public Health, 7938 University of Toronto, Toronto, ON, Canada

**Keywords:** aging, older adults, gay men, identity construction, intersectionality, grounded theory

## Abstract

Older gay men commonly conceal their sexual identity in healthcare settings due to past experiences and expectations of encountering stigma and discrimination in these contexts. Although insights on how older gay men construct their sexual identity in healthcare may help contextualize this phenomenon, this question remains under-explored. Accordingly, we present the findings of a secondary grounded theory analysis of individual interview data, which we originally collected to examine the healthcare experiences of 27 gay men ages 50 and over, to explore constructions of sexual identity among the group. Our findings broadly reveal that older gay men’s varying exposure to intersecting systems of oppression, together with their perceptions of different healthcare settings, may be critical in shaping their constructions of sexual identity in these contexts. Our research supports the need for healthcare policies and practices that address stigma and discrimination as salient barriers to sexual identity disclosure among older gay men.

## Introduction

There has been significant growth in scholarship on the healthcare experiences and needs of older gay men in recent years (Fredriksen-Goldsen et al., 2019). This research has substantiated the prominent exposure of this population to pervasive and pernicious expressions of stigma and discrimination, including those reflecting the intersection of homophobia and agism, across healthcare settings (Fredriksen-Goldsen et al., 2013; Zanetos & Skipper, 2020). Given this context, it is unsurprising that older gay men continue to face profound barriers to healthcare access, unmet healthcare needs, and a greater burden of poor physical and mental health outcomes relative to their heterosexual counterparts (Fredriksen-Goldsen et al., 2013; Lyons et al., 2019).

Recently, researchers have drawn attention to the potential relationship between sexual identity disclosure in healthcare, meaning openness about one’s sexual identity with healthcare providers, and improved healthcare experiences and health outcomes among sexual minorities (Bernstein et al., 2008; Cahill et al., 2014; Neville & Henrickson, 2006; Petroll & Mosack, 2011; Ramchand & Fox, 2008; Ruben & Fullerton, 2018). In a recent systematic review and meta-analysis in this area, Ruben and Fullerton (2018) found that sexual identity disclosure was related to higher satisfaction with healthcare services, greater levels of healthcare seeking and screening, and improved self-reported health and psychological well-being among lesbian, gay, and bisexual people. When sexual minority individuals lose a partner, sexual identity disclosure is often necessary for those surviving the loss to seek and receive relevant support from health professionals providing bereavement services (Bristowe et al., 2016). Often, a lack of disclosure is the result of gay men’s expectations and experiences of being mistreated upon revealing that they are gay. Given known fears surrounding disclosure among gay men who perceive and encounter cultural, religious, institutional, and other forms of anti-gay stigma in healthcare settings (Connolly & Lynch, 2016), there exists compelling empirical evidence for this conceptual link.

Interestingly, the limited literature on sexual identity disclosure among aging sexual minority adults has revealed that older gay men may be less likely than some of their younger counterparts (i.e., not including those younger than 25) to openly identify as gay men in their interactions with healthcare providers (Gardner et al., 2014; Trussler & Ham, 2016). This phenomenon has, in part, been attributed to the group’s generational exposure to particularly negative experiences with healthcare institutions prior to the proliferation of relevant human rights protections—and the emergence of more identity-affirming social norms surrounding sexual diversity (Sinković & Towler, 2018)—across many of the world’s industrialized regions (Gardner et al., 2014; Lyons et al., 2021). Some researchers have, in particular, described the role of profoundly homophobic and stigmatizing healthcare policies and practices during the height of the HIV/AIDS epidemic, many of which resulted in the systemic neglect and mistreatment of gay men living with and affected by the disease during this era, as being influential in shaping the healthcare expectations of older gay men today (Catalan et al., 2020; Rosenfeld et al., 2012). There has historically been a disproportionate burden of the HIV epidemic on specific populations of older gay men who are subjected to intersecting oppressions, including aging gay-identified men of color (Rosenfeld et al., 2012). Moreover, there remains a heightened contemporary exposure to stigma and discrimination among older gay men who are racialized and affected by HIV (Meanley et al., 2019). Taken together, these findings suggest that barriers to sexual identity disclosure may be particularly prominent among aging gay men affected by multiple, intersecting systems of oppression.

Despite the emergence of a small body of literature addressing the relative prominence of sexual identity non-disclosure among older gay men, little remains known about how older gay men perceive the relevance and meaning of disclosing their gay identity when they engage with healthcare systems and, in turn, how this reflexive process shapes their interactions with healthcare providers and other institutional actors in healthcare settings. The literature therefore continues to reflect a lack of insight on the diverse constructions of sexual identity among older gay men in the context of their experiences with healthcare systems. Scholarship addressing this gap could address questions regarding the variable salience of sexual identity across situations involving engagement with healthcare providers and institutions, and among diverse groups of older gay men differentiated by race, class, ability, and other factors. It could also generate an understanding of contextual factors influencing patterns of sexual identity disclosure and non-disclosure in this population’s healthcare experiences. Finally, such research could inform policy and programming aimed at mitigating the adverse systemic conditions that inhibit sexual identity disclosure, particularly in situations that older gay men believe might necessitate openness about one’s sexual identity. Although researchers have explored policy and programming implications of existing sexual identity disclosure research, including the need for measures that affirm sexual and gender minority identities (e.g., acknowledging sexual and gender minority patients in pamphlets) (Brooks et al., 2018), it is unclear whether older gay men would have distinctive needs in this area relative to their younger counterparts. In this article, we provide preliminary insight on how older gay men construct their sexual identities in the context of their engagement with healthcare systems. To do this, we conduct a secondary analysis of existing primary data, which we originally collected as part of a parent qualitative study about the healthcare experiences of older gay men, and use our analysis to address this question.

### Identity Construction and Translocational Intersectionality

Identity theorists have historically used the term “identity construction” to conceptualize identity as a working product of complex relational processes, with a salience and meaning (as a descriptor for one’s relationship with the social environment) that can and often does shift as a function of social context (Stryker & Burke, 2000). Scholars have referred to collective identity construction, relatedly, as the social processes through which subjects sharing socially intelligible experiences of “difference” from the dominant social order mobilize to cultivate fluid and ever-evolving communities of shared solidarity (Greenland & Taulke-Johnson, 2017; Polletta & Jasper, 2001). Though these communities may share a lexicon and be bound by common social and political aims, these features of belonging often fluctuate according to time and place (Polletta & Jasper, 2001).

Historical shifts in the meanings and uses of “gay,” as a term for some expressions of queerness, illustrate fluidity in the leveraging of collective identity as a vehicle for organizing and driving political change. Originally, gay lexicon served the discursive purpose of building solidarity in some sexual minorities who would draw on this language to “come out,” to develop activist communities in this process, and to draw on presumably shared experience among others identifying with this discourse to challenge socially and legally sanctioned anti-queer stigma (D’Emillio, 2002). However, Ghaziani (2011) has argued that as a result of profound changes in the social and political landscape of sexual minorities situated in the Western world since the 1990s, the adoption of highly salient and visible “gay identities” among these groups may be increasingly uncommon as a basis for community organizing and social action. Instead, in the “post-gay” era, sexual minorities may be more likely to take up discourses of “sameness” by adopting less prominent and differentiated sexual identities that reflect both a mainstreaming and depoliticization of same-gender sexuality, and that potentially privilege assimilationist (rather than activist) ends. Although researchers have seldom explored historically evolving meanings of gay identity among recipients of healthcare specifically, the likelihood of historical variation and fluidity is high in this context. To substantiate this possibility, we have already highlighted distinctions in older gay men’s healthcare expectations and experiences based on their generational exposure to particularly profound expressions of stigma and discrimination targeting their sexual identity (and often their HIV status) during the height of the HIV/AIDS epidemic (Rosenfeld et al., 2012), and thus their potential association of sexual identity disclosure with fear of mistreatment in these settings (Gardner et al., 2014).

In this study, we employ notions of identity construction, including collective identity construction, to attend to “gay identity” as a label whose relevance and meaning may fluctuate depending on the social context of different healthcare experiences, even while the term may be connected to a shared collective history among some sexual minority men. Using this lens, we specifically foreground variations in how gay identity is constructed in the healthcare narratives of the men in our study. Although the term “sexual orientation” is frequently invoked by participants in our research, in our own analysis we use the term “sexual identity” consistently to account for the fluid and often variable social construction of identifying as “gay” to claim and render visible same-gender sexual orientation among some sexual minority men (Suen et al., 2020).

Intersectionality, which serves as a complementary theoretical framework in our study, elucidates the role of intersecting systems of oppression in shaping the social conditions and experiences of multiply marginalized groups (Cho et al., 2013; Collins, 2019; Crenshaw, 1991; McCall, 2005). Originating in Black feminist scholarship and activism, intersectionality is increasingly adopted to conceptualize and interpret issues of sexual minority groups affected by interlocking forces of marginalization (Bowleg, 2008; Mink et al., 2014). These groups include older gay men, whose lives are often affected by their exposure to oppressive social processes involving the interplay of homophobia and heterosexism, agism, poverty, racism, and ableism, and other factors (Cronin & King, 2010; Meanley et al., 2019). Translocational analysis, which is sometimes used to frame intersectional inquiry (Anthias, 2008), highlights what is often the variable prominence of different facets of identity among subjects, particularly those affected by multiple systems of oppression, across different social contexts and situations. In other words, translocational analysis renders explicit the notion—often implied in intersectional thought—that different aspects of a subject’s identity may become more or less relevant in shaping their lived experience depending on the social construction of different situations. For example, a precariously employed cisgender heterosexual man may be impacted by the salience of his class-based marginalization in the workplace, where his positionality as a laborer is particularly relevant in shaping his experience, but prominently experience the privilege of his gendered social location while at home with his cisgender female partner (Anthias, 2014). Applying translocational analysis more explicitly to healthcare research, a middle class gay man may face the marginalization of his sexual identity profoundly in a publicly subsidized “mainstream” healthcare setting, particularly if he has experienced of stigma and discrimination in this context, but then prominently experience the privilege of his class position in paying privately for care in a long-term care facility specifically intended for gay men.

Given possible distinctions in the nature, frequency, and impacts of exposure to stigma and discrimination among older gay men who may be differentiated by race, class position, specific age, HIV status, ability, and other factors (Cronin & King, 2010; Meanley et al., 2019), we use a translocational frame to attend to the heightened or diminished salience of gay identity as it is constructed across older gay men’s healthcare accounts. In particular, as sexual identity disclosure is closely related to past experiences of stigma and discrimination (Brooks et al., 2018; Lyons et al., 2021), we draw on this lens to examine intersectional differences in older gay men’s experiences of marginalization—particularly as they relate to sexual identity construction—in the context of healthcare.

## Methods

We relied on constructivist approaches to grounded theory (Charmaz, 2006; Clarke & Charmaz, 2014) to inform our research design. As the primary objective of our parent study (Kia et al., 2019) originally included generating insight on the healthcare experiences of older gay men more generally, we initially incorporated aspects of situational analysis (Clarke, 2003) to develop a contextually rich grounded theory of healthcare as a site of subjugation and resistance among our participants. Following a preliminary analysis of our data, we recognized that participants provided in-depth insights on the salience and meaning of their gay identities, particularly when prompted to discuss the relevance of their sexual identity to their healthcare needs, and then applied a constructivist approach to grounded theory (Clarke & Charmaz, 2014) to undertake a secondary analysis of these accounts. To clarify, although we collected data on sexual identity from the start of our study, initially we did this to account for possible differences in healthcare experience that may be attributed to participants’ social location, and did not specifically focus our attention on analyzing varying processes of identity construction. It was through the current secondary analysis that we were able to further explore this area of inquiry. Below, we briefly review recruitment, sampling, and data collection of our parent study (Kia et al., 2019) on the healthcare experiences of older gay men, and then describe analytical procedures we used to arrive at insights we present in this article.

### Statement on Ethics

The study underwent review and approval by the University of Toronto’s HIV Research Ethics Board, and participants provided written informed consent prior to their involvement in the study. The participants were made aware, when initially providing their consent, that their data may be used to develop knowledge on a wide range of topics relating to older gay men’s engagement with healthcare systems.

### Recruitment and Sampling

We recruited 27 gay-identified men aged 50 or older through community organizations serving lesbian, gay, bisexual, transgender, queer, Two-Spirit, and other sexual and gender minorities (LGBTQ/2S+) in a large Canadian city. The age threshold we used to define older gay men (≥50) is common in research on the social conditions and experiences of older sexual and gender minority adults (Fredriksen-Goldsen et al., 2019). We summarize the demographic characteristics of participants in Supplementary Table 1.

Drawing on principles of theoretical sampling (Charmaz, 2006; Clarke & Charmaz, 2014), together with pre-existing insight on the potential role of HIV stigma, racism, and poverty in differentially impacting the healthcare experiences of diverse older gay men (Rosenfeld et al., 2012), our goal with sampling was initially to prioritize inclusion of participants living with HIV, and to maximize socioeconomic and racial variation in our sample. To this end, we were successful in recruiting a total of 16 men living with HIV into our study. While our incorporation of targeted recruitment and rigorous screening procedures enabled us to achieve socioeconomic heterogeneity in our sample, our participants were relatively homogeneous in relation to race. We maintained a commitment to theoretical sampling throughout the study, namely, by engaging in continual analysis of our emerging data to prioritize inclusion of additional groups of participants with perspectives that would have required greater attention and analysis. However, given the rich initial variability and eventual saturation of our data, we did not have to make adjustments to recruitment and screening procedures beyond those prioritizing the recruitment of gay men living with HIV, as well as those targeting diversity in socioeconomic status and race.

### Data Collection

We invited participants to take part in individual semi-structured interviews, averaging between 1–1.5 hours, in which we broadly asked them questions related to their experiences of seeking and receiving healthcare. At the start of each interview, we specifically asked participants to describe the meaning and relevance of their gay identity, together with the significance and salience of other aspects of their identity and lived experience, for their health issues and healthcare needs. These prompts surrounding identity, which we present for reference in Supplementary Table 2, resulted in the rich participant accounts on which we primarily base our current analysis. All participants were compensated with CAD $20 and reimbursed for public transportation, and were additionally provided with a list of relevant healthcare and social service resources in their area following each interview. We audio-recorded all interviews and took field notes after each of the encounters. We contracted transcriptionists to transcribe each of the interviews verbatim.

### Data Analysis

As mentioned above, the insights we present in this article are derived from existing primary data, which were collected as part of a parent study on the healthcare experiences of older gay men (Kia et al., 2019). Originally, we relied on situational analysis, which is a post-structuralist approach to grounded theory (Clarke, 2003), to construct a contextually rich account of our participants’ healthcare experiences. We have, since this time, recognized in-depth insights that participants shared regarding the salience and meaning of their gay identities, particularly in the context of their healthcare experiences, and have engaged in a secondary analysis of these primary data using a constructivist approach (Charmaz, 2006; Clarke & Charmaz, 2014). Secondary qualitative data analysis is considered appropriate when a dataset promises to address theoretical and substantive gaps in a body of literature that a researcher identifies during post hoc readings of the data (Dufour et al., 2019; Hinds et al., 1997; Whiteside et al., 2012).

We primarily focused our secondary data analysis on the responses that participants gave to the interview protocol items outlined in Supplementary Table 2, though we did analyze full transcripts as well in order to capture any relevant data appearing beyond responses to these questions. Following the tenets of constructivist grounded theory methods for secondary data analysis (Dufour et al., 2019; Whiteside et al., 2012), we led an inductive and iterative secondary analytical process to arrive at the article’s final insights. We began this process by first developing preliminary, descriptive open codes across participants’ responses to the interview questions under analysis, as well as any relevant text appearing elsewhere in interview transcripts. Through a process of constant comparison, specifically involving attention to common, higher order themes implied by clusters of open codes, we then developed axial codes that reflected participants’ insights on the variable salience and meaning of their sexual identity, as well as other aspects of their identity, in the context of their healthcare experiences. The final stage of data analysis involved a reflexive process of selective coding. This entailed cross-referencing axial codes against theoretical frameworks used to conceptualize the data (identity construction and translocational intersectionality) to extrapolate themes that most prominently addressed how older gay men construct their sexual identities in healthcare settings.

Although we were not able to incorporate theoretical sampling of participants to strengthen the rigor of our secondary analytical process, we accounted for this limitation by ensuring maximum variation in the range of participant accounts included for analysis, and by foregrounding distinctive or underrepresented participant insights in the process of selective coding. This alternative to theoretical sampling, which led to saturation of the insights we present in this article, has been successfully incorporated in other secondary data analyses of existing primary data involving the use of grounded theory methods (Whiteside et al., 2012).

## Findings

Our findings are broadly organized into four primary themes: (1) Practicing gay invisibility as a means of protection, (2) fearing the prospect of being “intelligibly” gay in specific healthcare contexts, (3) engaging in occasional disclosures of gay identity to purposefully address pragmatic and political ends, and (4) incorporating habitual, yet resistive disclosures of gay identity in the context of living with HIV. The insights reflected within each of these themes highlight the role of participants’ distinct social locations, and their resulting exposure to intersecting systemic forces targeting differences in sexuality, race, class position, ability, and HIV status, in shaping how they variably construct their gay identities across different healthcare situations and settings.

### Practicing Gay Invisibility as a Means of Protection

Several of our participants explained that maintaining an invisibility of their gay identities—whether by active non-disclosure or by their lack of objection to healthcare providers’ heteronormative assumptions—was a common practice for them when navigating healthcare settings. Explaining their reasons for this tendency, participants noted that they would often practice non-disclosure and other forms of invisibility to protect themselves against what they believed was a potential exposure to stigma and discrimination from healthcare providers. These accounts were particularly common among participants whose social locations reflected possible exposure to other intersecting forms of oppression such as racism, classism, and ableism, in addition to homophobia, heterosexism, and agism. For example, one of our participants, a Black African Canadian man in his 50s, explained that coming out to his family physician would entail compromising what he felt was his access to quality care:

Well coming out is a high-stakes situation depending on where you’re at … Why should I tell my doctor that I’m gay? … I don’t bring [up] my sexuality, because … it’s that issue of being judged, and is it really relevant?

When prompted to discuss factors that he believed made the prospect of disclosing his sexual identity particularly “high-stakes,” this participant added that he felt his access to care was especially tenuous because he was susceptible to racism as a Black man in healthcare settings, and did not want to jeopardize his access to a clinic that felt unusually comfortable for him:

Racism is alive and well, you step out there and you’re judged … [At my doctor’s clinic], I may be the only Black man in the room … but I feel at home right there.

Another participant, who was a white man in his 50s accessing disability benefits (which do not provide what would be considered a livable income in urban Canada [Canadian HIV/AIDS Legal Network, 2013]), described living with a number of mental health issues. He explained that although many of his recent healthcare experiences had been positive, he had also been in several encounters with healthcare providers whom he felt had mistreated him on the basis of his sexual identity, mental health, and class position. For example, he recalled an exchange with a psychiatrist in which he believed he was treated with hostility after discussing these aspects of his identity with the service provider:

So when I told him I was gay and had a handicap he was totally … like, you could tell I said the wrong things … I [also] have very little money and … he didn’t like social services [and people who use them].

Drawing on experiences like the above, this participant mentioned he was concerned about encountering homophobic stigma and discrimination in a residential care setting if he were to require this level of care in the future. He discussed perceiving the likelihood of experiencing stigma directed at his mental health—and at “being poor in a home”—as factors already complicating a possible transition to residential care and mentioned he would not want his gay identity visible as an additional target of discrimination. The participant noted that he would only be open to living in a facility if he were in such a “gay positive” institution that his sexual identity would, in some sense, be “invisible” to healthcare providers as a factor relevant to his care:

And, like I said, if I have to be, you know, in some sort of care home or residence it’s got to be completely gay positive … it [gay identity] would be invisible because it wouldn’t matter … I don’t want special treatment if that means bad treatment, just treat [me] normal, yeah.

While one participant actively practiced non-disclosure of his gay identity to maintain ties with a family physician that he felt was not prejudiced against him as a Black man, the other discussed preferences for accessing future care that would essentially negate the need for him to disclose his identity as a gay man. Both men appeared to construct their sexual identity as dimensions that they would ultimately want diminished in salience—in fact, to the point of invisibility—in order to manage their experiences with stigma and discrimination targeting what they believed were more intelligible aspects of their social location in healthcare settings.

### Fearing the Prospect of Being “Intelligibly” Gay in Specific Healthcare Contexts

Several of the men in our sample described fearing the prospect of being openly gay in particular healthcare settings and scenarios, namely, in residential care facilities and across situations involving contact with healthcare providers in the home. In the case of residential care, several participants noted worrying about the prospect of being stigmatized as gay men in institutional settings where they would potentially have limited agency in responding to mistreatment by healthcare providers. One white man in his 70s, who reported living exclusively on government-administered and means-tested old age security benefits, provided an account that clearly reflected this theme:

So what am I going to do if I’m in elder care and I am getting these silent messages ‘Oh this one’s a fag’ you know, ‘I think this one’s a fag, in bed two, I think he’s a fag’. What am I going to do?

This participant went on to rationalize his fear by explaining what he felt were his generation’s exposure to pathologizing discourses regarding gay identity, and the significant distress he had historically felt at the prospect of being psychiatrized or criminalized because of his sexual identity while in the care of an institution. Without being prompted, he also referred to his middle-class upbringing as a source of privilege that he feared losing if he were to render himself visible as a gay man in the context of any kind of institutionalized care:

In 1969, just as a reminder, [being gay] wasn’t only still a criminal act, it was in the psychiatric manual as a psychiatric disorder. And … for a long time I thought ‘I’m a good middle-class boy. I don’t want to be [labeled with a mental disorder] or a criminal [if I am ever in any kind of institutional care].

Several of the men reported experiencing and/or anticipating fear in having healthcare providers entering their homes. While some of these participants described engagement with homecare providers as a heuristic for such scenarios, others reflected on both the prospect and experience of being “intelligibly” gay in their contact with emergency responders. One example of the latter was reflected in our interview with a Filipino man in his 50s who was employed as a healthcare provider. This participant described having once called 911 during a health emergency and feeling “uncomfortable” over the possibility of being in a recognizably same-gender relationship and household to the emergency responders:

So we called 911, the people from the fire department got to me, the people got – an ambulance got to me. And at that point, because I realized it had nothing – my sexual orientation had nothing to do with [the emergency health issue], I never really spoke of it. And because there was all these straight men, right – I mean apparently straight men, apparently – fire fighters and medics coming at me all at once, you know, I didn’t feel comfortable having to talk about my sexual orientation … [yet] it was a bit obvious to them that we were gay because we were living in the same house and sharing the same bed.

For this participant, along with several others, situations involving home-based contact with healthcare providers often fueled fears about losing agency over their homes, which usually represented protection and reprieve from profound historical experiences of stigma and discrimination. This notion was particularly evident in the accounts of middle class participants, who had had (for the most part) lifelong access to safe and stable housing. The first participant quoted in this passage, along with several others, similarly discussed fears of being “intelligibly” gay in residential care settings, given their potentially diminished capacity to mitigate hostility and mistreatment directed at them in institutions that triggered troubled histories of the psychiatrization, criminalization, and normative oppression of gay identity. Together, these examples demonstrated how perceptions of certain healthcare contexts, particularly those symbolizing the encroachment of healthcare institutions on the living environment, may have represented particularly high risk situations for sexual identity disclosure among the participants. In these scenarios, the men constructed gay identity as a preferably hidden social location associated with significant vulnerability.

### Engaging in Occasional Disclosures of Gay Identity Purposefully to Address Pragmatic and Political Ends

Among HIV-negative men in our sample, many discussed eventually disclosing their sexual identities to long-time healthcare providers as a means of addressing specific healthcare needs, for example, those related to sexual health, even though initially they may have been greatly reluctant to do so. Such instances of disclosure represented rare, yet potentially irreversible occasions of gay intelligibility for the men, often after lengthy periods of concealment. These accounts were especially prominent among participants who held class privilege, including those who indicated they had access to employment income or private pensions, or who explicitly identified as middle class. One participant, a white man in his 50s who had retired a few years prior to the study from his career as a healthcare provider, explained that he had discussed his sexual identity with his family physician after years of concealing it from him, and did so only when he felt doing so was relevant to his primary care. He also explained that changes in social norms regarding same-gender sexuality were particularly critical in precipitating his comfort level with his gay identity, as well as his consequent disclosure, which (although irreversible in its impacts) was received well by his provider:

Well I think about how the world has changed and how society has evolved from the 80s to now. I mean, my coming out process was really tough for me. I started to experience the feelings when I was 16 but fought them and suppressed them [for many years] … When you look at my comfort level with myself, which is critical to my comfort level with my healthcare practitioner, it’s probably not that big a time gap. But again, I had to get comfortable with who I am … it wasn’t particularly relevant to discuss with my family doc [for a long time], and when it was, I did.

When prompted to describe the situation that had triggered this participant to disclose his sexual identity to his family physician, he noted that he had raised a sexual health issue that he believed would have required revealing he had a male partner. He also mentioned that while he did not feel disclosing his sexuality was relevant in many acute medical situations, he believed conditions enabling such disclosure were important for the provision of humanizing healthcare:

Generally speaking, sexual orientation is not relevant. It’s relevant in terms of the holistic approach and, you know, [the idea that] the patient is more than just the sick person in front of you … And it’s relevant when I’m an inpatient in the hospital and my husband is spending time with me. Those are the scenarios where it’s particularly relevant.

Perhaps in contrast to the above participant’s narrative, some of the other HIV-negative men in our sample acknowledged that while they had rarely disclosed their sexual identities to healthcare providers, they were prepared to do so as a means of resisting the conditions of homophobia and heterosexism that they felt were systemically reflected across healthcare settings. For example, one white man in his 70s, who also identified as middle class, mentioned that he would render his gay identity visible in a residential care facility if he were to ever require such care, and would specifically do so to challenge narratives of older gay men “going back into the closet.” He referenced generational norms associated counterculture (“we are the flower children”) in explaining his position:

But this whole idea of what happens and elderly people going back into the closet … we are the flower children [and] we’re not going back into the closet … Why am I raising this? It had to do with sort of looking in the future and elder care and so on … People like me are going to say ‘Move over honey, I’m going to tell you the story [of how] I did drag for 15 years and I was hot’. And I was hot.

Whereas one participant indicated a tendency to limit discussing his gay identity unless he believed doing so was relevant to his care, the other felt rendering himself visible—specifically as a politically resistive (albeit hypothetical) practice—was important in some healthcare settings. Despite these differences, both of the men described disclosures of gay identity as being uncommon and often done to address specific needs. Unlike participants who commented on the protective function of rendering invisible their sexual identities, many of whom were affected by multiple, intersecting systems of oppression, the two men described in this section (white, middle class, and presumably able-bodied) practiced self-disclosure selectively, but did so to meet specific ends. These men constructed their sexual identity, in other words, as a dimension of social location that could be rendered visible to address distinctive healthcare issues and needs, but also acknowledged the risks of doing so.

### Incorporating Habitual, Yet Resistive Disclosures of Gay Identity in the Context of Living with HIV

In contrast to the relatively uncommon disclosures of gay identity among HIV-negative men in our sample, gay men living with HIV generally reported regularly disclosing their sexual identity to healthcare providers. In most cases, the men had become accustomed to openly discussing their sexual identities after receiving specialized HIV treatment services intended (at least originally) for gay men, some of which had been created as alternatives to “mainstream” care early in the history of the disease. For example, one white man in his 50s, who had been living with HIV since the 1980s, explained that when he was first diagnosed, he was referred to a specialty clinic that he recalled had—in response to stigma surrounding treatment of gay men living with HIV in “mainstream” settings—been exclusively developed to treat those in this group:

That’s when my medical needs changed, the family doctor could no longer take care of me because back then they didn’t know anything at all. They were not educated with the AIDS virus at all so they couldn’t treat anybody. That’s why I was put in a study at [a specialty clinic] … [This clinic] started treating gay men because the community [didn’t want others involved] with the AIDS virus.

In situations where the men did not describe longstanding experiences with specialized HIV treatment intended for gay men, they still referred to what they believed was the regularity of gay men accessing HIV specialty clinics in explaining their relative comfort with disclosure in these settings. Another white man living with HIV, who was also in his 50s, provided an account that reflected this notion:

The people I’m meeting [in HIV clinics] are so specific to working and treating people just like me … you know, for the longest time, it was just gay men.

Interestingly, among gay men living with HIV, disclosures of sexual identity were not only highly frequent in the context of the participants’ interactions with healthcare providers, but they were often additionally motivated by their resistance to what they perceived to be homophobic and stigmatizing practices. Our interview with a white man in his 60s, who had been living with HIV for more than 10 years, contained an excerpt that clearly illustrated this theme. The participant discussed having met with a homecare provider whom he believed had incorporated excessive contact precautions while in his home, which he attributed to the provider’s potential prejudice against gay men living with HIV. Although he believed that the homecare organization had already obtained information on his gay identity and HIV status from his primary care team, which he thought had led to the homecare provider’s excessive precautions, he nonetheless discussed being gay and HIV-positive during the encounter, and then leveraged these aspects of his identity to demand better care from the organization.

[I said] you’re putting on a Hazmat suit! You know I’m gay, you know I’m HIV positive, this is ridiculous … And if your reason for doing it is because [I’m gay and HIV positive] I’m sending you away … I couldn’t accept somebody going in, and every time they come in, go “oh, is it going to be okay?” … I [called the organization and] said I sent them away, can you send somebody else today please?

Accounts such as that of this participant were present in nearly all of our interviews with gay men who were living with HIV, which demonstrated both the regularity and intent with which participants in this group would disclose and/or claim their gay identities in healthcare settings. This segment of our sample appeared to construct gay identity as a highly salient feature of lived experience that had, potentially due to the historical relationship of gay men with the HIV epidemic (Rosenfeld et al., 2012), become inextricably linked with their HIV diagnosis as an intelligible target of stigma and discrimination. They also, not surprisingly, constructed gay identity as a site of resistance against the marginalizing practices they had come to expect and experience, based on past and ongoing experiences with healthcare providers. It is important to note that some men in this group described positive and affirming experiences in their experiences with healthcare professionals, but even these participants discussed anticipating the need to occasionally challenge stigma and discrimination and expressed a readiness to do so if necessary.

## Discussion

In this article, we drew on existing qualitative data to examine how older gay men construct gay identity in their encounters with service providers and other actors in healthcare settings. The four themes we constructed illustrate that the men ascribed variable salience and meaning to their sexual identities, depending on factors such as their social location, their perception of certain healthcare settings as being particularly hostile to gay men, and their HIV status. Together, our findings revealed that constructions of gay identity, for older gay men, may be highly heterogeneous, and may fluctuate based on a complex interplay of social location (meaning the positioning of the self, relative to sexual identity, race, class, ability, HIV status, and other intersecting axes of power) and social context (meaning one’s understanding of a specific social environment). This interpretation of our findings is consistent with theories of identity construction (Polletta & Jasper, 2001; Stryker & Burke, 2000) and translocational intersectionality (Anthias, 2008). While the former supports the notion of identity as a descriptor whose relevance and meaning can fluctuate significantly based on social processes involving the subject’s engagement with their social context (Stryker & Burke, 2000), the latter foregrounds the role of socially intelligible systems of power in shaping context-dependent differences in the salience and construction of specific identities (Anthias, 2008).

As constructions of gay identity, among our participants, were intimately connected with their perceptions of—and experiences with—sexual identity disclosure in healthcare settings, we situate our findings substantively within the emerging body of scholarship on sexual identity disclosure. There now exists a body of literature concerning the significance of sexual identity disclosure among gay men, irrespective of age, as a site of critical healthcare research (Brooks et al., 2018; Gardner et al., 2014). Our study’s findings corroborate the importance of inquiry on this topic as they elucidate the relationship between disclosure and quality care. Indeed, in their constructions of sexual identity, several of the men described situations that they felt necessitated sexual identity disclosure as a condition of receiving appropriate and humanized care, which is a theme that other scholars have foregrounded in the literature on disclosure (Bristowe et al., 2016; Ramchand & Fox, 2008).

Despite the significance of disclosure for the provision of healthcare, the literature in this area has highlighted that older gay men often experience unique barriers to sexual identity disclosure relative to their younger counterparts (Gardner et al., 2014). These barriers include older gay men’s expectations of mistreatment by healthcare providers, which may in part be connected with this population’s negative experiences navigating profoundly stigma-laden healthcare systems at the height of HIV/AIDS epidemic (Gardner et al., 2014; Trussler & Ham, 2016). Our findings support the premise that the historical context of HIV/AIDS may be particularly salient in influencing sexual identity disclosure among older gay men, yet also foreground variation and complexity surrounding this theoretical picture. For example, while participants living with HIV experienced their gay identity (inseparably of their HIV status) as a highly intelligible dimension of their social location in healthcare settings, primarily due to the historical “pairing” of HIV care with gay men’s health (Rosenfeld et al., 2012), HIV-negative men experienced agency over rendering themselves intelligibly gay in situations involving specific pragmatic and political ends. Some men in our sample, particularly those affected by multiple systems of oppression, discussed the protective functions of rendering their gay identities invisible, which would have served as an added target of discrimination if revealed to healthcare providers. Finally, the men in our sample identified some healthcare settings, namely, those representing the encroachment of healthcare systems on the home, as being particularly “high stakes” contexts for practicing disclosure, and thus often expressed preferences for limiting their visibility as gay men in these settings.

Our study may be used to inform future inquiry on issues of identity and disclosure among sexual minorities, including older gay men, particularly as they relate to sexual minority health. First, our study substantiates the need for research on disclosure patterns across different groups of older gay men and other sexual minorities. Scholars have, indeed, highlighted the important role of sexual identity disclosure in shaping the health outcomes and healthcare experiences of sexual minorities (Brooks et al., 2018; Panchakis, 2007; Panchakis et al., 2020; Ruben & Fullerton, 2018). Drawing on this notion, we believe researchers could leverage our insights on variability in perceptions and experiences of disclosure to examine distinctions in disclosure patterns across sexual minority groups differentiated by age, specific sexual identity, race, ability, class position, and healthcare context, as well as the impacts of these differences on healthcare experiences and health outcomes. They could then utilize these insights, in collaboration with community and professional stakeholders, to refine healthcare policies and practices designed to facilitate safer sexual identity disclosure where appropriate, or to mitigate harms associated with non-disclosure (or post-disclosure discrimination) as necessary.

Beyond substantive issues in the area of sexual identity disclosure, our study also promises to contribute to the scholarship on identity construction (Greenland & Taulke-Johnson, 2017; Stryker & Burke, 2000) and translocational approaches to intersectionality (Anthias, 2008, 2014). As already noted, our study yields insights that are consistent with the premises of both bodies of scholarship, primarily by drawing attention to sexual identity among older gay men as a complex relational product of the interplay between social location and social context. Accordingly, our research substantiates the applicability of identity construction and translocational frames for conceptualizing and conducting future inquiry on the variable salience and meaning of sexual identity among sexual minorities, both in healthcare contexts and in other domains. Recent contributions to intersectional queer scholarship have, indeed, drawn attention to the possibility that a mostly homogenous, salient, and stable “gay identity” is diminishing in relevance, due in part to changes in social norms and other sociopolitical factors related to the emancipation of sexual minorities in many industrialized regions of the world (Ghaziani, 2011; Greenland & Taulke-Johnson, 2017). While our study supports the notion that the salience and meaning of gay identity may increasingly be varied and context-dependent, it also highlights the prominently risk-ridden nature of gay intelligibility for certain age categories of gay men, as well as gay men whose social locations reflect exposure to multiple systems of oppression. Our study, additionally, foregrounds the significance of gay identity as an intelligible dimension of difference for older gay-identified men living with HIV, many of whom continue to be affected by the sociohistorical legacy of receiving care in highly homophobic and stigma-laden healthcare systems of the early HIV/AIDS epidemic. These are subjects who have established collective activist identities from the early days of the HIV epidemic and, in so doing, have contributed to the resistance of stigma and discrimination in healthcare (Rosenfeld et al., 2012). These insights further the scholarship on identity construction and translocational intersectionality by substantiating their utility in rendering contextually rich and nuanced accounts of identity among groups affected by complex and distinctive histories of marginalization.

Several implications of our study for healthcare policy and practice merit consideration. First, we believe policies and position statements clarifying institutional commitments to addressing stigma and discrimination against sexual minorities—which would then be made visible in the form of signage, visual displays, and patient education materials—are critically important for creating conditions of enhanced inclusion for these populations. Given that many of the men in our sample described entering healthcare settings with expectations of mistreatment, which would then impede on their capacity to practice sexual identity disclosure, we believe such work could function to improve the trust of older sexual minorities (including older gay men) in health institutions vis à vis treatment of their sexual identities. Relatedly, our findings reveal the importance of training healthcare providers on the historically adverse conditions to which older gay men and other sexual minorities have been exposed in healthcare settings, and on practices that could serve to mitigate the impacts of this history. Several of the men reported interpreting healthcare providers’ behaviors as signaling discomfort with their gay identity and/or HIV status, often as a result of their past experiences with healthcare systems, and would then conclude that their sexual identities continue to be targets of stigma and discrimination in contemporary healthcare. Practices that affirm non-heterosexuality, including the use of gender-neutral language to refer to patients’ spouses (“partner” as opposed to “wife” or “husband”), along with providing information on resources for sexual and gender minorities to all patients (regardless of assumptions about their social location), may be some strategies to promote in such training.

In closing our article, it is important to note some of our study’s limitations. As noted, we were unable to recruit a large number of racialized older gay men, which may have impacted the range of insights we were able to generate about the intersecting role of racism in shaping identity construction among those in this population. Given preliminary insights we highlighted in this study, we encourage future researchers to address this important gap in their work. Moreover, our research took place in a large urban center in Canada and therefore may lack transferability to other contexts, including rural regions, as well as less industrialized regions of the world where older gay men face different forms of stigmatization. While these limitations will warrant attention in future studies, our work represents an important contribution to studies of sexual identity among older gay men and other sexual minorities, and we hope that our consideration of these shortcomings can serve to catalyze opportunities for other researchers to contribute to this emerging body of scholarship. Given ongoing changes in the salience and meaning of gay identity, across social locations and disparate social contexts (Ghaziani, 2011), this area of work will remain pertinent—and will likely continue raising questions—for social and health scientists in the foreseeable future.

## Supplemental Material

sj-pdf-1-qhr-10.1177_10497323211050373 – Supplemental Material for “You Could Tell I Said the Wrong Things”: Constructions of Sexual Identity Among Older Gay Men in Healthcare Settings Supplemental Material, sj-pdf-1-qhr-10.1177_10497323211050373 for “You Could Tell I Said the Wrong Things”: Constructions of Sexual Identity Among Older Gay Men in Healthcare Settings by Hannah Kia, Travis Salway, Ashley Lacombe-Duncan, Olivier Ferlatte and Lori E. Ross in Qualitative Health Research
