# Supplementary material for: “You Could Tell I Said the Wrong Things”: Constructions of Sexual Identity Among Older Gay Men in Healthcare Settings
Source: Qual Health Res. 2021 Dec 7;32(2):255–66. doi: 10.1177/10497323211050373 (PMC8727827; doi:10.1177/10497323211050373)
Supplement: sj-pdf-1-qhr-10.1177_10497323211050373 – Supplemental Material for “You Could Tell I Said the Wrong Things”: Constructions of Sexual Identity Among Older Gay Men in Healthcare Settings [file sj-pdf-1-qhr-10.1177_10497323211050373.pdf]

Supplementary Table 1 – Demographic Characteristics of Participants

		HIV-Positive Subsample (n=16)	HIV-Negative Subsample (n=11)
**Age**	Ages 50-59	11	4
	Ages 60-69	4	2
	Ages 70+	1	5
**Racial/Ethnic Identity**	White	13	9
	Black/Afro-Caribbean/African	1	1
	Other (Latin, Asian/Pacific Islander, Mixed Race)	2	1
**Socioeconomic Status**	Exclusively reliant on government-administered financial benefits for income	11	2
	Access to employment income, private pensions, and other non-governmental sources of income	5	9

Supplementary Table 2 – Interview Protocol Items of Interest for Secondary Analysis

1. Can you tell me what it means for you to identify as a gay man?<br>a. How relevant do you think your sexual identity is when it comes to your health care needs?
2. Other than your sexual identity, are there aspects of your identity or experiences, for example your age, HIV status, ethnicity, race, mental health status, addiction history, gender, class background, or other aspects that you feel are relevant or important to mention when you think about your experiences of accessing services?<br>a. How relevant do you think these other aspects of your identity are when it comes to your health care needs?<br>b. When you think about the health settings you're in most frequently, to what extent do you feel like you're able to bring your whole self to these settings? Are there ever pieces of yourself or your identity that don't seem to fit in these settings?
